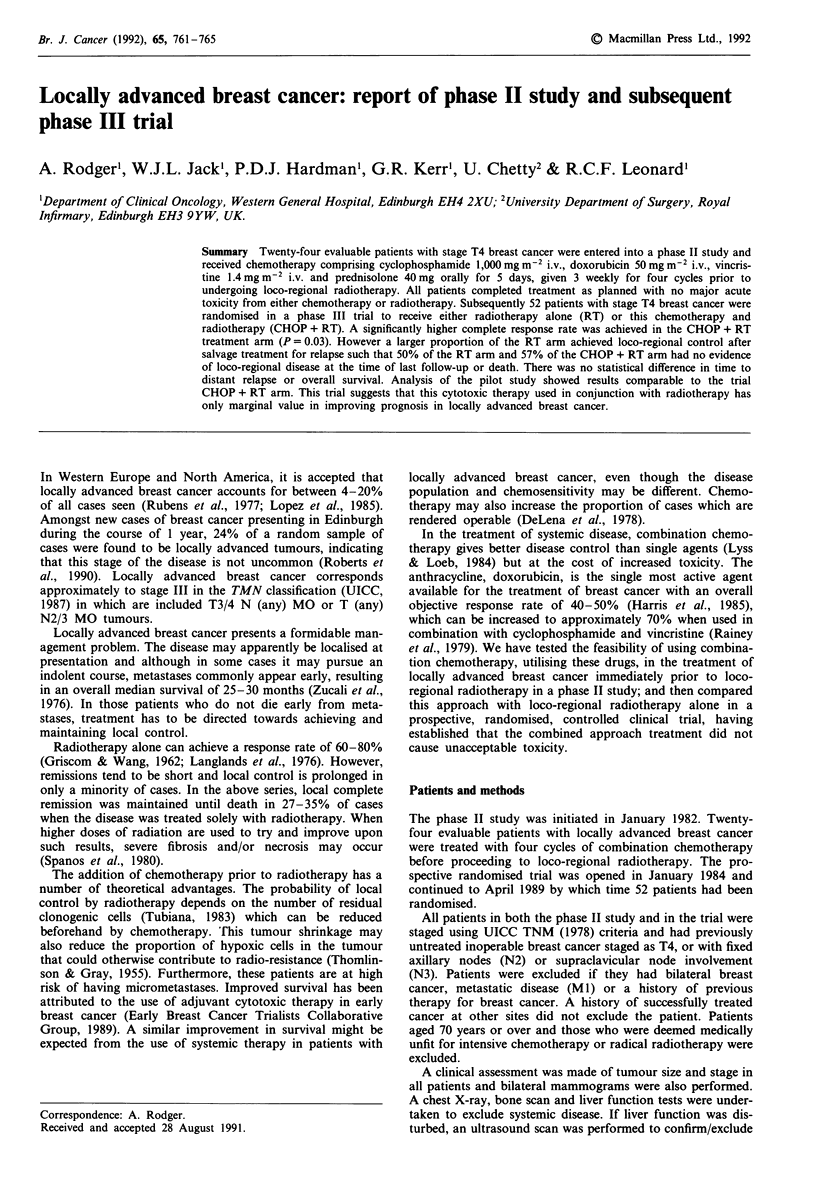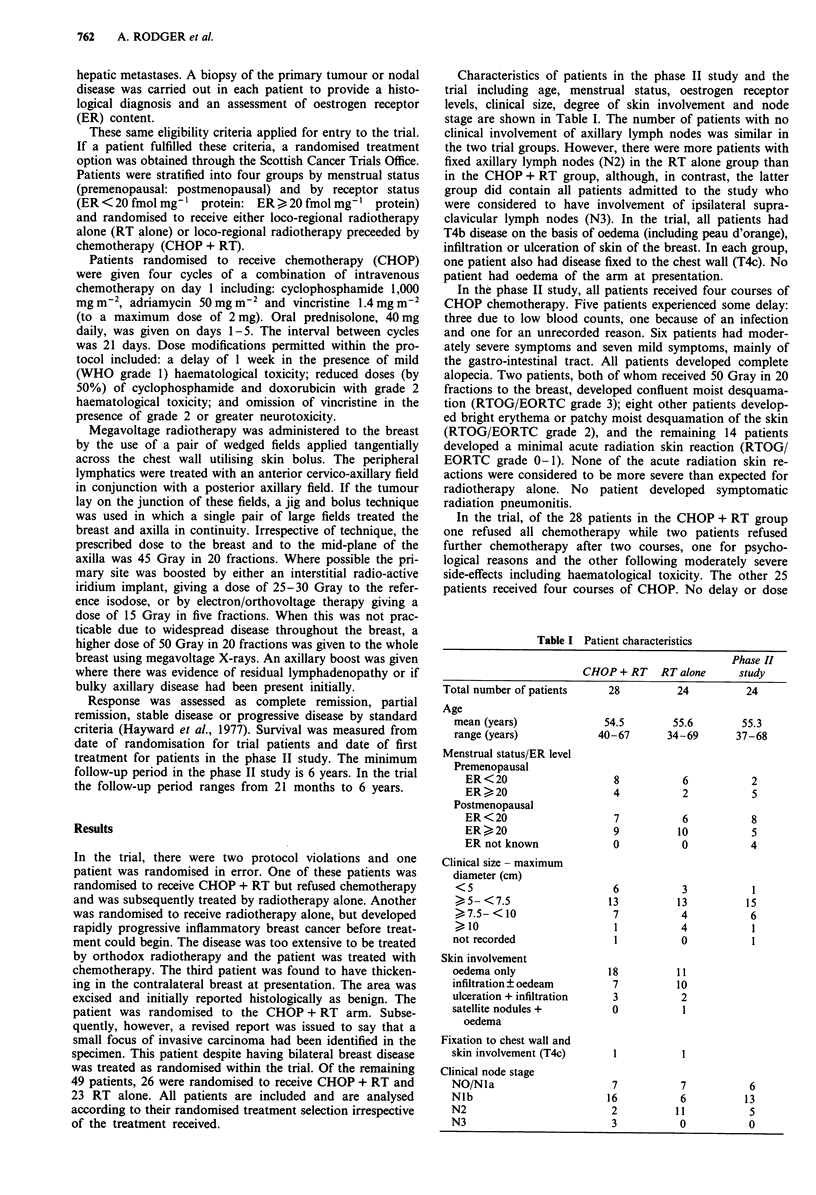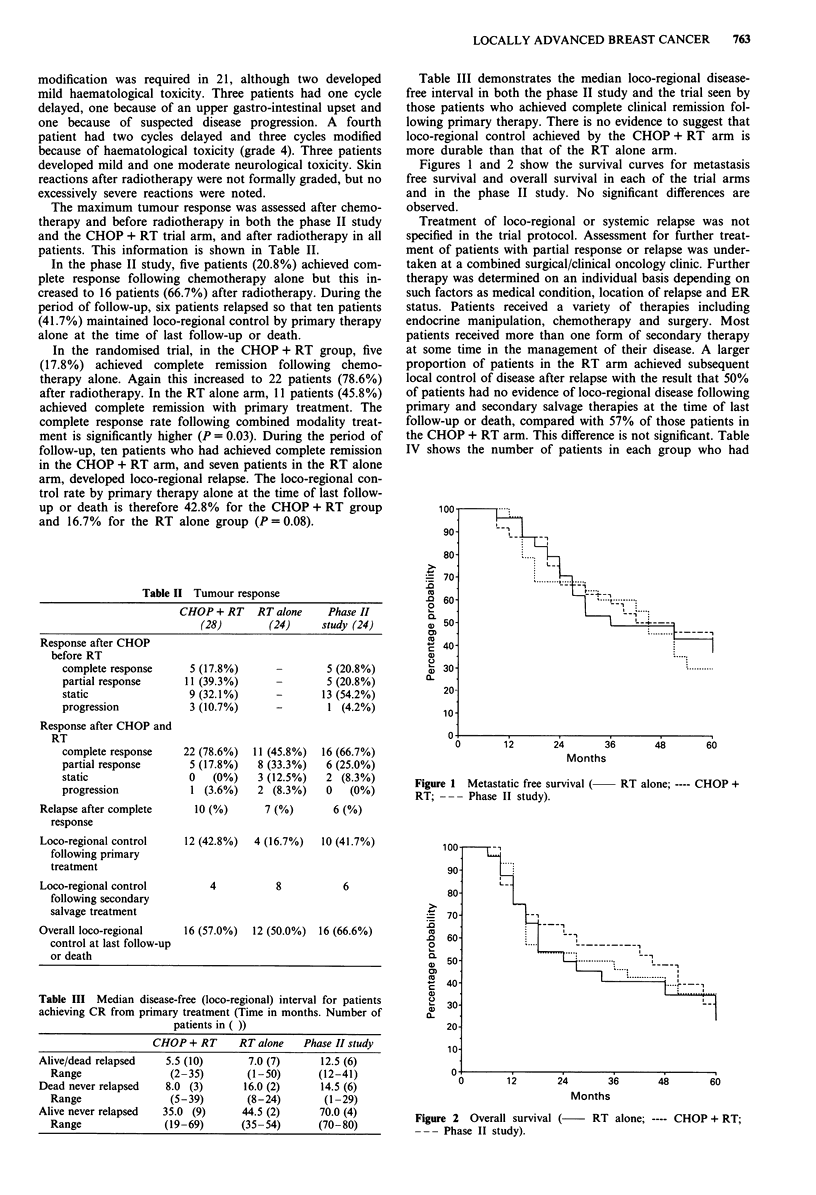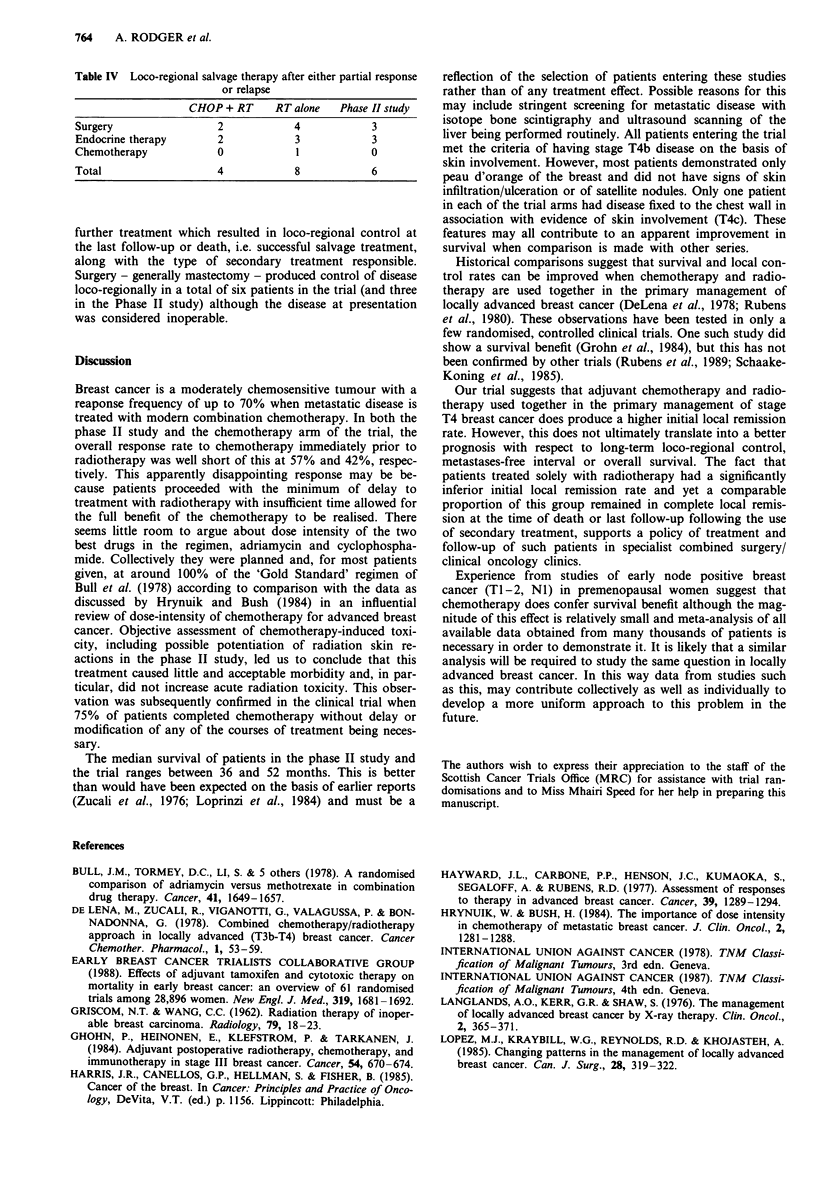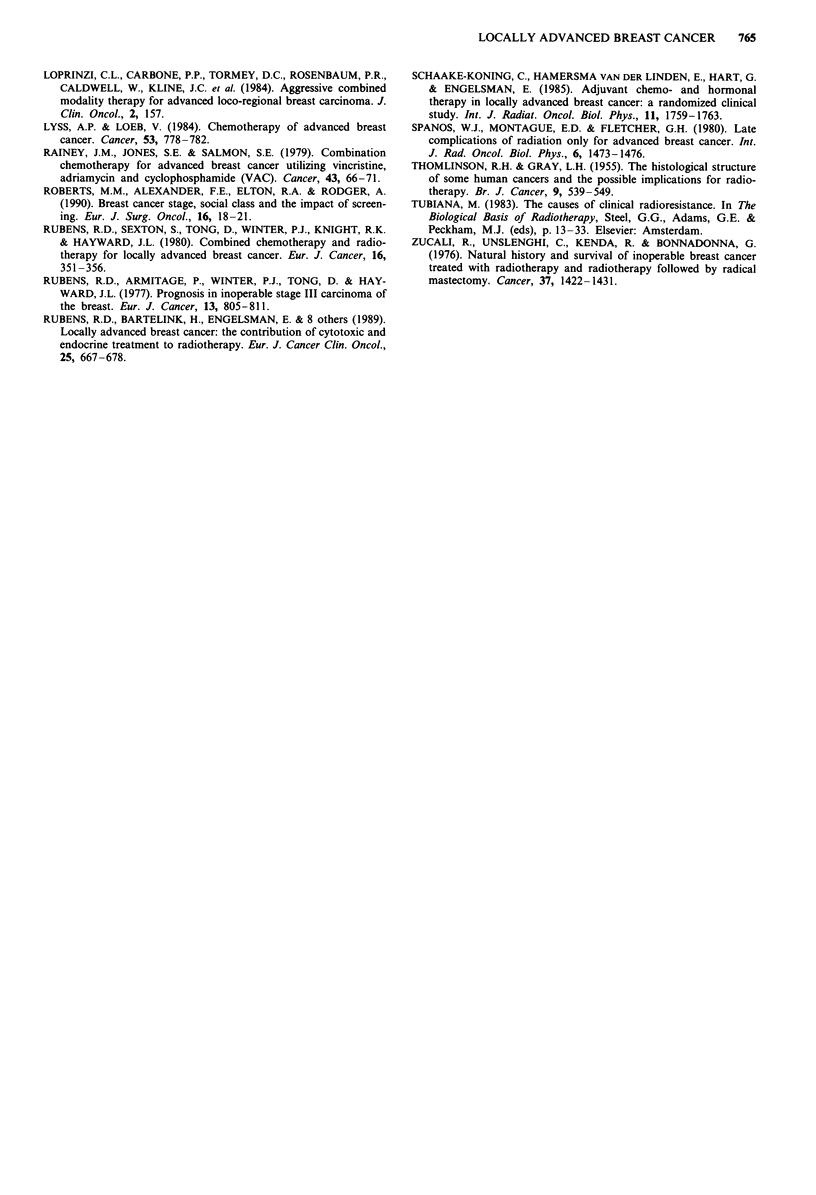# Locally advanced breast cancer: report of phase II study and subsequent phase III trial.

**DOI:** 10.1038/bjc.1992.160

**Published:** 1992-05

**Authors:** A. Rodger, W. J. Jack, P. D. Hardman, G. R. Kerr, U. Chetty, R. C. Leonard

**Affiliations:** Department of Clinical Oncology, Western General Hospital, Edinburgh, UK.

## Abstract

Twenty-four evaluable patients with stage T4 breast cancer were entered into a phase II study and received chemotherapy comprising cyclophosphamide 1,000 mg m-2 i.v., doxorubicin 50 mg m-2 i.v., vincristine 1.4 mg m-2 i.v. and prednisolone 40 mg orally for 5 days, given 3 weekly for four cycles prior to undergoing loco-regional radiotherapy. All patients completed treatment as planned with no major acute toxicity from either chemotherapy or radiotherapy. Subsequently 52 patients with stage T4 breast cancer were randomised in a phase III trial to receive either radiotherapy alone (RT) or this chemotherapy and radiotherapy (CHOP + RT). A significantly higher complete response rate was achieved in the CHOP + RT treatment arm (P = 0.03). However a larger proportion of the RT arm achieved loco-regional control after salvage treatment for relapse such that 50% of the RT arm and 57% of the CHOP + RT arm had no evidence of loco-regional disease at the time of last follow-up or death. There was no statistical difference in time to distant relapse or overall survival. Analysis of the pilot study showed results comparable to the trial CHOP + RT arm. This trial suggests that this cytotoxic therapy used in conjunction with radiotherapy has only marginal value in improving prognosis in locally advanced breast cancer.


*Br. J. Cancer* (1992), **65**, 761–765 © Macmillan Press Ltd., 1992

### Locally advanced breast cancer: report of phase II study and subsequent phase III trial

A. Rodger[1], W.J.L. Jack[1], P.D.J. Hardman[1], G.R. Kerr[1], U. Chetty[2] & R.C.F. Leonard[1]

[1]*Department of Clinical Oncology, Western General Hospital, Edinburgh EH4 2XU;* [2]*University Department of Surgery, Royal Infirmary, Edinburgh EH3 9YW, UK.*

**Summary** Twenty-four evaluable patients with stage T4 breast cancer were entered into a phase II study and received chemotherapy comprising cyclophosphamide 1,000 mg m$^{-2}$ i.v., doxorubicin 50 mg m$^{-2}$ i.v., vincristine 1.4 mg m$^{-2}$ i.v. and prednisolone 40 mg orally for 5 days, given 3 weekly for four cycles prior to undergoing loco-regional radiotherapy. All patients completed treatment as planned with no major acute toxicity from either chemotherapy or radiotherapy. Subsequently 52 patients with stage T4 breast cancer were randomised in a phase III trial to receive either radiotherapy alone (RT) or this chemotherapy and radiotherapy (CHOP + RT). A significantly higher complete response rate was achieved in the CHOP + RT treatment arm ($P = 0.03$). However a larger proportion of the RT arm achieved loco-regional control after salvage treatment for relapse such that 50% of the RT arm and 57% of the CHOP + RT arm had no evidence of loco-regional disease at the time of last follow-up or death. There was no statistical difference in time to distant relapse or overall survival. Analysis of the pilot study showed results comparable to the trial CHOP + RT arm. This trial suggests that this cytotoxic therapy used in conjunction with radiotherapy has only marginal value in improving prognosis in locally advanced breast cancer.

In Western Europe and North America, it is accepted that locally advanced breast cancer accounts for between 4–20% of all cases seen (Rubens *et al.*, 1977; Lopez *et al.*, 1985). Amongst new cases of breast cancer presenting in Edinburgh during the course of 1 year, 24% of a random sample of cases were found to be locally advanced tumours, indicating that this stage of the disease is not uncommon (Roberts *et al.*, 1990). Locally advanced breast cancer corresponds approximately to stage III in the *TMN* classification (UICC, 1987) in which are included T3/4 N (any) MO or T (any) N2/3 MO tumours.

Locally advanced breast cancer presents a formidable management problem. The disease may apparently be localised at presentation and although in some cases it may pursue an indolent course, metastases commonly appear early, resulting in an overall median survival of 25–30 months (Zucali *et al.*, 1976). In those patients who do not die early from metastases, treatment has to be directed towards achieving and maintaining local control.

Radiotherapy alone can achieve a response rate of 60–80% (Griscom & Wang, 1962; Langlands *et al.*, 1976). However, remissions tend to be short and local control is prolonged in only a minority of cases. In the above series, local complete remission was maintained until death in 27–35% of cases when the disease was treated solely with radiotherapy. When higher doses of radiation are used to try and improve upon such results, severe fibrosis and/or necrosis may occur (Spanos *et al.*, 1980).

The addition of chemotherapy prior to radiotherapy has a number of theoretical advantages. The probability of local control by radiotherapy depends on the number of residual clonogenic cells (Tubiana, 1983) which can be reduced beforehand by chemotherapy. This tumour shrinkage may also reduce the proportion of hypoxic cells in the tumour that could otherwise contribute to radio-resistance (Thomlinson & Gray, 1955). Furthermore, these patients are at high risk of having micrometastases. Improved survival has been attributed to the use of adjuvant cytotoxic therapy in early breast cancer (Early Breast Cancer Trialists Collaborative Group, 1989). A similar improvement in survival might be expected from the use of systemic therapy in patients with locally advanced breast cancer, even though the disease population and chemosensitivity may be different. Chemotherapy may also increase the proportion of cases which are rendered operable (DeLena *et al.*, 1978).

In the treatment of systemic disease, combination chemotherapy gives better disease control than single agents (Lyss & Loeb, 1984) but at the cost of increased toxicity. The anthracycline, doxorubicin, is the single most active agent available for the treatment of breast cancer with an overall objective response rate of 40–50% (Harris *et al.*, 1985), which can be increased to approximately 70% when used in combination with cyclophosphamide and vincristine (Rainey *et al.*, 1979). We have tested the feasibility of using combination chemotherapy, utilising these drugs, in the treatment of locally advanced breast cancer immediately prior to loco-regional radiotherapy in a phase II study; and then compared this approach with loco-regional radiotherapy alone in a prospective, randomised, controlled clinical trial, having established that the combined approach treatment did not cause unacceptable toxicity.

### Patients and methods

The phase II study was initiated in January 1982. Twenty-four evaluable patients with locally advanced breast cancer were treated with four cycles of combination chemotherapy before proceeding to loco-regional radiotherapy. The prospective randomised trial was opened in January 1984 and continued to April 1989 by which time 52 patients had been randomised.

All patients in both the phase II study and in the trial were staged using UICC TNM (1978) criteria and had previously untreated inoperable breast cancer staged as T4, or with fixed axillary nodes (N2) or supraclavicular node involvement (N3). Patients were excluded if they had bilateral breast cancer, metastatic disease (M1) or a history of previous therapy for breast cancer. A history of successfully treated cancer at other sites did not exclude the patient. Patients aged 70 years or over and those who were deemed medically unfit for intensive chemotherapy or radical radiotherapy were excluded.

A clinical assessment was made of tumour size and stage in all patients and bilateral mammograms were also performed. A chest X-ray, bone scan and liver function tests were undertaken to exclude systemic disease. If liver function was disturbed, an ultrasound scan was performed to confirm/exclude

Correspondence: A. Rodger.
Received and accepted 28 August 1991.

hepatic metastases. A biopsy of the primary tumour or nodal disease was carried out in each patient to provide a histological diagnosis and an assessment of oestrogen receptor (ER) content.

These same eligibility criteria applied for entry to the trial. If a patient fulfilled these criteria, a randomised treatment option was obtained through the Scottish Cancer Trials Office. Patients were stratified into four groups by menstrual status (premenopausal: postmenopausal) and by receptor status (ER $<$ 20 fmol mg$^{-1}$ protein: ER $\geqslant$ 20 fmol mg$^{-1}$ protein) and randomised to receive either loco-regional radiotherapy alone (RT alone) or loco-regional radiotherapy preceeded by chemotherapy (CHOP + RT).

Patients randomised to receive chemotherapy (CHOP) were given four cycles of a combination of intravenous chemotherapy on day 1 including: cyclophosphamide 1,000 mg m$^{-2}$, adriamycin 50 mg m$^{-2}$ and vincristine 1.4 mg m$^{-2}$ (to a maximum dose of 2 mg). Oral prednisolone, 40 mg daily, was given on days 1–5. The interval between cycles was 21 days. Dose modifications permitted within the protocol included: a delay of 1 week in the presence of mild (WHO grade 1) haematological toxicity; reduced doses (by 50%) of cyclophosphamide and doxorubicin with grade 2 haematological toxicity; and omission of vincristine in the presence of grade 2 or greater neurotoxicity.

Megavoltage radiotherapy was administered to the breast by the use of a pair of wedged fields applied tangentially across the chest wall utilising skin bolus. The peripheral lymphatics were treated with an anterior cervico-axillary field in conjunction with a posterior axillary field. If the tumour lay on the junction of these fields, a jig and bolus technique was used in which a single pair of large fields treated the breast and axilla in continuity. Irrespective of technique, the prescribed dose to the breast and to the mid-plane of the axilla was 45 Gray in 20 fractions. Where possible the primary site was boosted by either an interstitial radio-active iridium implant, giving a dose of 25–30 Gray to the reference isodose, or by electron/orthovoltage therapy giving a dose of 15 Gray in five fractions. When this was not practicable due to widespread disease throughout the breast, a higher dose of 50 Gray in 20 fractions was given to the whole breast using megavoltage X-rays. An axillary boost was given where there was evidence of residual lymphadenopathy or if bulky axillary disease had been present initially.

Response was assessed as complete remission, partial remission, stable disease or progressive disease by standard criteria (Hayward *et al.*, 1977). Survival was measured from date of randomisation for trial patients and date of first treatment for patients in the phase II study. The minimum follow-up period in the phase II study is 6 years. In the trial the follow-up period ranges from 21 months to 6 years.

### Results

In the trial, there were two protocol violations and one patient was randomised in error. One of these patients was randomised to receive CHOP + RT but refused chemotherapy and was subsequently treated by radiotherapy alone. Another was randomised to receive radiotherapy alone, but developed rapidly progressive inflammatory breast cancer before treatment could begin. The disease was too extensive to be treated by orthodox radiotherapy and the patient was treated with chemotherapy. The third patient was found to have thickening in the contralateral breast at presentation. The area was excised and initially reported histologically as benign. The patient was randomised to the CHOP + RT arm. Subsequently, however, a revised report was issued to say that a small focus of invasive carcinoma had been identified in the specimen. This patient despite having bilateral breast disease was treated as randomised within the trial. Of the remaining 49 patients, 26 were randomised to receive CHOP + RT and 23 RT alone. All patients are included and are analysed according to their randomised treatment selection irrespective of the treatment received.

Characteristics of patients in the phase II study and the trial including age, menstrual status, oestrogen receptor levels, clinical size, degree of skin involvement and node stage are shown in Table I. The number of patients with no clinical involvement of axillary lymph nodes was similar in the two trial groups. However, there were more patients with fixed axillary lymph nodes (N2) in the RT alone group than in the CHOP + RT group, although, in contrast, the latter group did contain all patients admitted to the study who were considered to have involvement of ipsilateral supraclavicular lymph nodes (N3). In the trial, all patients had T4b disease on the basis of oedema (including peau d'orange), infiltration or ulceration of skin of the breast. In each group, one patient also had disease fixed to the chest wall (T4c). No patient had oedema of the arm at presentation.

In the phase II study, all patients received four courses of CHOP chemotherapy. Five patients experienced some delay: three due to low blood counts, one because of an infection and one for an unrecorded reason. Six patients had moderately severe symptoms and seven mild symptoms, mainly of the gastro-intestinal tract. All patients developed complete alopecia. Two patients, both of whom received 50 Gray in 20 fractions to the breast, developed confluent moist desquamation (RTOG/EORTC grade 3); eight other patients developed bright erythema or patchy moist desquamation of the skin (RTOG/EORTC grade 2), and the remaining 14 patients developed a minimal acute radiation skin reaction (RTOG/EORTC grade 0–1). None of the acute radiation skin reactions were considered to be more severe than expected for radiotherapy alone. No patient developed symptomatic radiation pneumonitis.

In the trial, of the 28 patients in the CHOP + RT group one refused all chemotherapy while two patients refused further chemotherapy after two courses, one for psychological reasons and the other following moderately severe side-effects including haematological toxicity. The other 25 patients received four courses of CHOP. No delay or dose

**Table I** Patient characteristics

| | *CHOP + RT* | *RT alone* | *Phase II study* |
|---|---|---|---|
| Total number of patients | 28 | 24 | 24 |
| Age | | | |
| mean (years) | 54.5 | 55.6 | 55.3 |
| range (years) | 40–67 | 34–69 | 37–68 |
| Menstrual status/ER level | | | |
| Premenopausal | | | |
| ER $<$ 20 | 8 | 6 | 2 |
| ER $\geqslant$ 20 | 4 | 2 | 5 |
| Postmenopausal | | | |
| ER $<$ 20 | 7 | 6 | 8 |
| ER $\geqslant$ 20 | 9 | 10 | 5 |
| ER not known | 0 | 0 | 4 |
| Clinical size – maximum diameter (cm) | | | |
| $<$ 5 | 6 | 3 | 1 |
| $\geqslant$ 5– $<$ 7.5 | 13 | 13 | 15 |
| $\geqslant$ 7.5– $<$ 10 | 7 | 4 | 6 |
| $\geqslant$ 10 | 1 | 4 | 1 |
| not recorded | 1 | 0 | 1 |
| Skin involvement | | | |
| oedema only | 18 | 11 | |
| infiltration ± oedeam | 7 | 10 | |
| ulceration + infiltration | 3 | 2 | |
| satellite nodules + oedema | 0 | 1 | |
| Fixation to chest wall and skin involvement (T4c) | 1 | 1 | |
| Clinical node stage | | | |
| NO/N1a | 7 | 7 | 6 |
| N1b | 16 | 6 | 13 |
| N2 | 2 | 11 | 5 |
| N3 | 3 | 0 | 0 |

modification was required in 21, although two developed mild haematological toxicity. Three patients had one cycle delayed, one because of an upper gastro-intestinal upset and one because of suspected disease progression. A fourth patient had two cycles delayed and three cycles modified because of haematological toxicity (grade 4). Three patients developed mild and one moderate neurological toxicity. Skin reactions after radiotherapy were not formally graded, but no excessively severe reactions were noted.

The maximum tumour response was assessed after chemotherapy and before radiotherapy in both the phase II study and the CHOP + RT trial arm, and after radiotherapy in all patients. This information is shown in Table II.

In the phase II study, five patients (20.8%) achieved complete response following chemotherapy alone but this increased to 16 patients (66.7%) after radiotherapy. During the period of follow-up, six patients relapsed so that ten patients (41.7%) maintained loco-regional control by primary therapy alone at the time of last follow-up or death.

In the randomised trial, in the CHOP + RT group, five (17.8%) achieved complete remission following chemotherapy alone. Again this increased to 22 patients (78.6%) after radiotherapy. In the RT alone arm, 11 patients (45.8%) achieved complete remission with primary treatment. The complete response rate following combined modality treatment is significantly higher ($P = 0.03$). During the period of follow-up, ten patients who had achieved complete remission in the CHOP + RT arm, and seven patients in the RT alone arm, developed loco-regional relapse. The loco-regional control rate by primary therapy alone at the time of last follow-up or death is therefore 42.8% for the CHOP + RT group and 16.7% for the RT alone group ($P = 0.08$).

**Table II** Tumour response

| | *CHOP + RT (28)* | *RT alone (24)* | *Phase II study (24)* |
|---|---|---|---|
| Response after CHOP before RT | | | |
| complete response | 5 (17.8%) | – | 5 (20.8%) |
| partial response | 11 (39.3%) | – | 5 (20.8%) |
| static | 9 (32.1%) | – | 13 (54.2%) |
| progression | 3 (10.7%) | – | 1 (4.2%) |
| Response after CHOP and RT | | | |
| complete response | 22 (78.6%) | 11 (45.8%) | 16 (66.7%) |
| partial response | 5 (17.8%) | 8 (33.3%) | 6 (25.0%) |
| static | 0 (0%) | 3 (12.5%) | 2 (8.3%) |
| progression | 1 (3.6%) | 2 (8.3%) | 0 (0%) |
| Relapse after complete response | 10 (%) | 7 (%) | 6 (%) |
| Loco-regional control following primary treatment | 12 (42.8%) | 4 (16.7%) | 10 (41.7%) |
| Loco-regional control following secondary salvage treatment | 4 | 8 | 6 |
| Overall loco-regional control at last follow-up or death | 16 (57.0%) | 12 (50.0%) | 16 (66.6%) |

**Table III** Median disease-free (loco-regional) interval for patients achieving CR from primary treatment (Time in months. Number of patients in ( ))

| | *CHOP + RT* | *RT alone* | *Phase II study* |
|---|---|---|---|
| Alive/dead relapsed | 5.5 (10) | 7.0 (7) | 12.5 (6) |
| Range | (2–35) | (1–50) | (12–41) |
| Dead never relapsed | 8.0 (3) | 16.0 (2) | 14.5 (6) |
| Range | (5–39) | (8–24) | (1–29) |
| Alive never relapsed | 35.0 (9) | 44.5 (2) | 70.0 (4) |
| Range | (19–69) | (35–54) | (70–80) |

Table III demonstrates the median loco-regional disease-free interval in both the phase II study and the trial seen by those patients who achieved complete clinical remission following primary therapy. There is no evidence to suggest that loco-regional control achieved by the CHOP + RT arm is more durable than that of the RT alone arm.

Figures 1 and 2 show the survival curves for metastasis free survival and overall survival in each of the trial arms and in the phase II study. No significant differences are observed.

Treatment of loco-regional or systemic relapse was not specified in the trial protocol. Assessment for further treatment of patients with partial response or relapse was undertaken at a combined surgical/clinical oncology clinic. Further therapy was determined on an individual basis depending on such factors as medical condition, location of relapse and ER status. Patients received a variety of therapies including endocrine manipulation, chemotherapy and surgery. Most patients received more than one form of secondary therapy at some time in the management of their disease. A larger proportion of patients in the RT arm achieved subsequent local control of disease after relapse with the result that 50% of patients had no evidence of loco-regional disease following primary and secondary salvage therapies at the time of last follow-up or death, compared with 57% of those patients in the CHOP + RT arm. This difference is not significant. Table IV shows the number of patients in each group who had

**Figure 1** Metastatic free survival (—— RT alone; ---- CHOP + RT; – – – Phase II study).

**Figure 2** Overall survival (—— RT alone; ---- CHOP + RT; – – – Phase II study).

**Table IV** Loco-regional salvage therapy after either partial response or relapse

| | *CHOP + RT* | *RT alone* | *Phase II study* |
|---|---|---|---|
| Surgery | 2 | 4 | 3 |
| Endocrine therapy | 2 | 3 | 3 |
| Chemotherapy | 0 | 1 | 0 |
| Total | 4 | 8 | 6 |

further treatment which resulted in loco-regional control at the last follow-up or death, i.e. successful salvage treatment, along with the type of secondary treatment responsible. Surgery – generally mastectomy – produced control of disease loco-regionally in a total of six patients in the trial (and three in the Phase II study) although the disease at presentation was considered inoperable.

### Discussion

Breast cancer is a moderately chemosensitive tumour with a reaponse frequency of up to 70% when metastatic disease is treated with modern combination chemotherapy. In both the phase II study and the chemotherapy arm of the trial, the overall response rate to chemotherapy immediately prior to radiotherapy was well short of this at 57% and 42%, respectively. This apparently disappointing response may be because patients proceeded with the minimum of delay to treatment with radiotherapy with insufficient time allowed for the full benefit of the chemotherapy to be realised. There seems little room to argue about dose intensity of the two best drugs in the regimen, adriamycin and cyclophosphamide. Collectively they were planned and, for most patients given, at around 100% of the 'Gold Standard' regimen of Bull *et al.* (1978) according to comparison with the data as discussed by Hrynuik and Bush (1984) in an influential review of dose-intensity of chemotherapy for advanced breast cancer. Objective assessment of chemotherapy-induced toxicity, including possible potentiation of radiation skin reactions in the phase II study, led us to conclude that this treatment caused little and acceptable morbidity and, in particular, did not increase acute radiation toxicity. This observation was subsequently confirmed in the clinical trial when 75% of patients completed chemotherapy without delay or modification of any of the courses of treatment being necessary.

The median survival of patients in the phase II study and the trial ranges between 36 and 52 months. This is better than would have been expected on the basis of earlier reports (Zucali *et al.*, 1976; Loprinzi *et al.*, 1984) and must be a reflection of the selection of patients entering these studies rather than of any treatment effect. Possible reasons for this may include stringent screening for metastatic disease with isotope bone scintigraphy and ultrasound scanning of the liver being performed routinely. All patients entering the trial met the criteria of having stage T4b disease on the basis of skin involvement. However, most patients demonstrated only peau d'orange of the breast and did not have signs of skin infiltration/ulceration or of satellite nodules. Only one patient in each of the trial arms had disease fixed to the chest wall in association with evidence of skin involvement (T4c). These features may all contribute to an apparent improvement in survival when comparison is made with other series.

Historical comparisons suggest that survival and local control rates can be improved when chemotherapy and radiotherapy are used together in the primary management of locally advanced breast cancer (DeLena *et al.*, 1978; Rubens *et al.*, 1980). These observations have been tested in only a few randomised, controlled clinical trials. One such study did show a survival benefit (Grohn *et al.*, 1984), but this has not been confirmed by other trials (Rubens *et al.*, 1989; Schaake-Koning *et al.*, 1985).

Our trial suggests that adjuvant chemotherapy and radiotherapy used together in the primary management of stage T4 breast cancer does produce a higher initial local remission rate. However, this does not ultimately translate into a better prognosis with respect to long-term loco-regional control, metastases-free interval or overall survival. The fact that patients treated solely with radiotherapy had a significantly inferior initial local remission rate and yet a comparable proportion of this group remained in complete local remission at the time of death or last follow-up following the use of secondary treatment, supports a policy of treatment and follow-up of such patients in specialist combined surgery/clinical oncology clinics.

Experience from studies of early node positive breast cancer (T1–2, N1) in premenopausal women suggest that chemotherapy does confer survival benefit although the magnitude of this effect is relatively small and meta-analysis of all available data obtained from many thousands of patients is necessary in order to demonstrate it. It is likely that a similar analysis will be required to study the same question in locally advanced breast cancer. In this way data from studies such as this, may contribute collectively as well as individually to develop a more uniform approach to this problem in the future.

The authors wish to express their appreciation to the staff of the Scottish Cancer Trials Office (MRC) for assistance with trial randomisations and to Miss Mhairi Speed for her help in preparing this manuscript.

### References

BULL, J.M., TORMEY, D.C., LI, S. & 5 others (1978). A randomised comparison of adriamycin versus methotrexate in combination drug therapy. *Cancer*, **41**, 1649–1657.

DE LENA, M., ZUCALI, R., VIGANOTTI, G., VALAGUSSA, P. & BONNADONNA, G. (1978). Combined chemotherapy/radiotherapy approach in locally advanced (T3b-T4) breast cancer. *Cancer Chemother. Pharmacol.*, **1**, 53–59.

EARLY BREAST CANCER TRIALISTS COLLABORATIVE GROUP (1988). Effects of adjuvant tamoxifen and cytotoxic therapy on mortality in early breast cancer: an overview of 61 randomised trials among 28,896 women. *New Engl. J. Med.*, **319**, 1681–1692.

GRISCOM, N.T. & WANG, C.C. (1962). Radiation therapy of inoperable breast carcinoma. *Radiology*, **79**, 18–23.

GHOHN, P., HEINONEN, E., KLEFSTROM, P. & TARKANEN, J. (1984). Adjuvant postoperative radiotherapy, chemotherapy, and immunotherapy in stage III breast cancer. *Cancer*, **54**, 670–674.

HARRIS, J.R., CANELLOS, G.P., HELLMAN, S. & FISHER, B. (1985). Cancer of the breast. In *Cancer: Principles and Practice of Oncology*, DeVita, V.T. (ed.) p. 1156. Lippincott: Philadelphia.

HAYWARD, J.L., CARBONE, P.P., HENSON, J.C., KUMAOKA, S., SEGALOFF, A. & RUBENS, R.D. (1977). Assessment of responses to therapy in advanced breast cancer. *Cancer*, **39**, 1289–1294.

HRYNUIK, W. & BUSH, H. (1984). The importance of dose intensity in chemotherapy of metastatic breast cancer. *J. Clin. Oncol.*, **2**, 1281–1288.

INTERNATIONAL UNION AGAINST CANCER (1978). *TNM Classification of Malignant Tumours*, 3rd edn. Geneva.

INTERNATIONAL UNION AGAINST CANCER (1987). *TNM Classification of Malignant Tumours*, 4th edn. Geneva.

LANGLANDS, A.O., KERR, G.R. & SHAW, S. (1976). The management of locally advanced breast cancer by X-ray therapy. *Clin. Oncol.*, **2**, 365–371.

LOPEZ, M.J., KRAYBILL, W.G., REYNOLDS, R.D. & KHOJASTEH, A. (1985). Changing patterns in the management of locally advanced breast cancer. *Can. J. Surg.*, **28**, 319–322.

LOPRINZI, C.L., CARBONE, P.P., TORMEY, D.C., ROSENBAUM, P.R., CALDWELL, W., KLINE, J.C. *et al.* (1984). Aggressive combined modality therapy for advanced loco-regional breast carcinoma. *J. Clin. Oncol.*, **2**, 157.

LYSS, A.P. & LOEB, V. (1984). Chemotherapy of advanced breast cancer. *Cancer*, **53**, 778–782.

RAINEY, J.M., JONES, S.E. & SALMON, S.E. (1979). Combination chemotherapy for advanced breast cancer utilizing vincristine, adriamycin and cyclophosphamide (VAC). *Cancer*, **43**, 66–71.

ROBERTS, M.M., ALEXANDER, F.E., ELTON, R.A. & RODGER, A. (1990). Breast cancer stage, social class and the impact of screening. *Eur. J. Surg. Oncol.*, **16**, 18–21.

RUBENS, R.D., SEXTON, S., TONG, D., WINTER, P.J., KNIGHT, R.K. & HAYWARD, J.L. (1980). Combined chemotherapy and radiotherapy for locally advanced breast cancer. *Eur. J. Cancer*, **16**, 351–356.

RUBENS, R.D., ARMITAGE, P., WINTER, P.J., TONG, D. & HAYWARD, J.L. (1977). Prognosis in inoperable stage III carcinoma of the breast. *Eur. J. Cancer*, **13**, 805–811.

RUBENS, R.D., BARTELINK, H., ENGELSMAN, E. & 8 others (1989). Locally advanced breast cancer: the contribution of cytotoxic and endocrine treatment to radiotherapy. *Eur. J. Cancer Clin. Oncol.*, **25**, 667–678.

SCHAAKE-KONING, C., HAMERSMA VAN DER LINDEN, E., HART, G. & ENGELSMAN, E. (1985). Adjuvant chemo- and hormonal therapy in locally advanced breast cancer: a randomized clinical study. *Int. J. Radiat. Oncol. Biol. Phys.*, **11**, 1759–1763.

SPANOS, W.J., MONTAGUE, E.D. & FLETCHER, G.H. (1980). Late complications of radiation only for advanced breast cancer. *Int. J. Rad. Oncol. Biol. Phys.*, **6**, 1473–1476.

THOMLINSON, R.H. & GRAY, L.H. (1955). The histological structure of some human cancers and the possible implications for radiotherapy. *Br. J. Cancer*, **9**, 539–549.

TUBIANA, M. (1983). The causes of clinical radioresistance. In *The Biological Basis of Radiotherapy*, Steel, G.G., Adams, G.E. & Peckham, M.J. (eds), p. 13–33. Elsevier: Amsterdam.

ZUCALI, R., UNSLENGHI, C., KENDA, R. & BONNADONNA, G. (1976). Natural history and survival of inoperable breast cancer treated with radiotherapy and radiotherapy followed by radical mastectomy. *Cancer*, **37**, 1422–1431.